# Impact of energy metabolism pathways in promoting phytoremediation of cadmium contamination by *Bacillus amyloliquefaciens* Bam1

**DOI:** 10.1186/s40643-025-00972-8

**Published:** 2025-11-10

**Authors:** Xinting Jiang, Xiaomin Chen, Hongxia Gao, Jinyan Luo, Lin Zhang, Yuanchan Luo, Hui Wu

**Affiliations:** 1 State Key Laboratory of Bioreactor Engineering, Shanghai Collaborative Innovation Center for Biomanufacturing Technology, School of Biotechnology, East China University of Science and Technology, 130 Meilong Road, Shanghai, 200237 China; 2 Department of Plant Quarantine, Shanghai Extension and Service Center of Agriculture Technology, Shanghai, 201103 China; 3 School of Chemistry and Molecular Engineering and Research Centre of Analysis and Test, East China University of Science and Technology, Shanghai, 200237 China; 4 MOE Key Laboratory of Bio-Intelligent Manufacturing, School of Bioengineering, Dalian University of Technology, Dalian, 116024 China

**Keywords:** Cadmium pollution remediation, Transcriptome, Energy metabolism pathway, *Bacillus amyloliquefaciens*

## Abstract

Remediation plants combined with plant growth promoting rhizobacteria (PGPR) is one of the most promising means of remediation of Cd-contaminated soils at present. One of the PGPR, named *Bacillus amyloliquefaciens* Bam1, possessed high Cd resistance. Herein, comparative transcriptome analysis of *B. amyloliquefaciens* Bam1 revealed that its main energy metabolism pathway was significantly down-regulated under Cd stress. The pivotal genes involved in the energy production pathway, such as TCA cycle and respiratory chain, were then selected to construct the energy production enhanced strains named as Bam1*sdhA*, Bam1*fumC*, and Bam1*qoxD*. The Cd resistance of the three recombinant strains increased significantly by producing more ATP and less ROS, allowing them to colonize Cd-contaminated soil better than the wild-type Bam1 strain. The better colonization of strain Bam1*fumC* improved the photosynthesis and growth of the remediation plant, tomatoes, under Cd stress significantly. Furthermore, the Cd concentration accumulated in tomatoes with the Cd + Bam1*fumC* treatment was 1.88 times that of the Cd + Bam1 treatment. As the energy production enhanced, Bam1*fumC* exhibited considerable potential for development as a bioaugmentation assistant in Cd-contaminated phytoremediation. This study also provided a novel strategy for addressing soil Cd pollution remediation.

**Graphical abstract:**

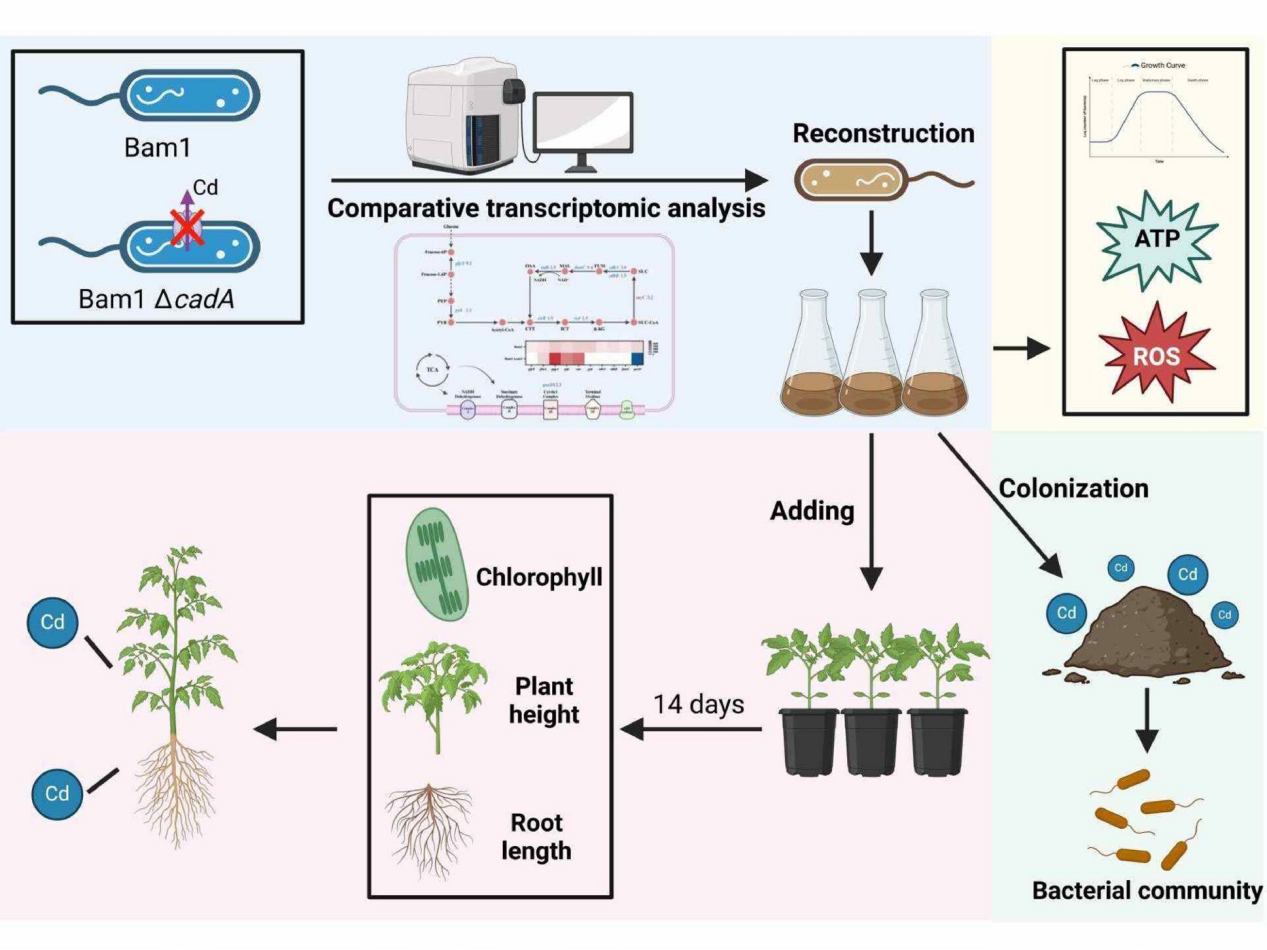

**Supplementary Information:**

The online version contains supplementary material available at 10.1186/s40643-025-00972-8.

## Introduction

The extensive industrial, mining, and agricultural activities have caused heavy metal contamination in soil, posing a significant worldwide environmental pollution issue (Hou et al. 2020; Ibitoye et al. 2024). Cadmium (Cd) is a major metal pollutant that poses a grave threat to agricultural soil and ecosystems (Zhang et al. 2022). It can inhibit plant growth by suppressing photosynthetic activity, restricting root elongation and plant height, and it even causes irreversible impairment to human via the food chain (Dary et al. 2010; Rai et al. 2019; Zhao et al. 2025a, b). Various physical and chemical techniques have been developed to immobilize, detoxify, or remove Cd from agricultural soil (Wang et al. 2021a). Alternatively, bioremediation by combining plant and PGPR (plant growth promoting rhizobacteria) is a more cost-effective, environmentally friendly, and sustainable strategy for Cd remediation (Danyal et al. 2023; Fu et al. 2024; Ouyang et al. 2025). It has been reported that PGPR can promote plant growth under Cd stress and further immobilize Cd, facilitating phytostabilization of Cd in soil (Ke et al. 2021; Li et al. 2022; Nie et al. 2023).

The ability to colonize and reproduce effectively in application habitats is essential for PGPR to promote plant growth (Wang et al. 2024). Therefore, when PGPR is utilized in Cd-contaminated soil, it necessitates greater Cd resistance, enabling it to reproduce a substantial population to aid in the growth of phytoremediation plants (Tiwari et al. 2025). Current research on microbial promotion of plant Cd remediation primarily focuses on how microorganisms facilitate alterations in plant metabolism and morphology to stimulate plant growth and Cd absorption, while research on enhancing the Cd resistance of PGPR and subsequently boosting the efficiency of plant remediation has been less extensive (Mao et al. 2022; Qiao et al. 2024; Wang et al. 2021b). The reported PGPR species used in Cd pollution bioremediation include *Bacillus*, *Pseudomonas*, *Azospirillum*, *Burkholderia*, *Streptomyces*, etc. (Bravo and Braissant 2022; Zulfiqar et al. 2022).

Traditionally, influx pumps, efflux pumps, and metal-sensitive regulatory proteins are considered crucial mechanisms that contribute to PGPR’s resistance against heavy metals (Chandrangsu et al. 2017; He et al. 2023; Niu et al. 2024; Sachla et al. 2021). Regarding microorganisms’ resistance to Cd, it is important to note that Cd is not an essential element for any organism. Therefore, microorganisms lack specific influx pumps for Cd. However, Cd^2+^ can still enter cells through other divalent ion channels, such as the major influx pump MntH, which is responsible for Mg^2+^ import in *Bacillus subtilis* (Huang et al. 2017; Moore and Helmann 2005). Reducing the expression of MntH can decrease the entry of Cd into *B. subtilis* (Moore and Helmann 2005)*.* The Cd-specific efflux pump, CadA (a P-type ATPase), is considered a vital mechanism for Cd resistance in many species (including *B. subtilis*, *B. amyloliquefaciens, Stenotrophomonas maltophilia*, *P. putida*, and *Helicobacter pylori*), and is regulated by CadC or its homolog (such as CzrA in *Bacillus*) (Luo et al. 2022; Sharma et al. 2024b).

The P-type ATPase efflux system, CadA, requires energy from ATP hydrolysis to transport metals, which is mainly for effluxing Cd but also other metals such as Fe and Mn (Prabhakaran and Thamarai 2024). Therefore, energy is hypothesized to be an important factor influencing microbial resistance to heavy metals including Cd. An efficient way to produce energy is to combine the central carbon metabolic pathways (e.g. glycolysis, tricarboxylic acid cycle (TCA), etc.) with the respiratory chain, which also generates toxic reactive oxygen species (ROS) as by-products (An et al. 2025; Han et al. 2023; Yan et al. 2024). Excessive ROS can also be generated by heavy metals for their redox catalysis function, causing damage to DNA, proteins, and lipids, ultimately leading to cell death (Abd Elnabi et al. 2023). A few studies have proved that generating sufficient energy significantly impacts the heavy metal resistance of microorganisms (Li et al. 2017; Tarrant et al. 2019). For example, in a mechanistic study of a *Staphylococcus aureus* strain against copper (Cu) stress, Tarrant et al. mention that the bacterium adapts to this stress by altering its central carbon metabolic pathways (Tarrant et al. 2019). When *S. aureus* was cultured in rich media with excess exogenous Cu, the key genes in the glycolysis pathway, *gapA* (encoding glyceraldehyde-3-phosphate dehydrogenase) and *fadA* (encoding fructose-bisphosphate aldolase), were significantly induced, and then more energy was produced to cope with the Cu stress (Tarrant et al. 2019). While another remediation microorganism, *Agrobacterium tumefaciens* GW4 strain, is activated to produce more energy for Sb transport and DNA repair via dissimilatory respiration, thereby increasing resistance to Sb^3+^ (Li et al. 2017). However, studies on the impact of energy metabolism on microbial Cd resistance have been less reported. Among the published studies on microbial Cd resistance, Khan et al. found that under Cd stress, a significant decrease in carbon metabolism was detected by proteomic analysis, indicating that energy is a critical factor in Cd resistance (Khan et al. 2017). In a study on bioremediation of hybrid *Pennisetum* under combined stress from polypropylene microplastics and Cd, by metagenomics analysis, Zhao et al. revealed that energy metabolism, including the pentose phosphate pathway, was an important mechanism for alleviating Cd stress in PGPR (Zhao et al. 2025a, b).

Based on previous research, we hypothesized that by appropriately enhancing the energy production pathway in a remediating PGPR, it would produce more energy to cope with Cd stress. The simultaneous production of reduced ROS by this modified PGPR strain will further facilitate its Cd resistance, thereby assisting its colonization and reproduction in Cd-contaminated niches. Moreover, the effective colonization of the modified PGPR can promote the growth of plants subjected to Cd stress, thereby enhancing their capacity for Cd remediation. A PGPR strain with demonstrated resistance to Cd, *B. amyloliquefaciens* Bam1 (Luo et al. 2022), was selected for the subsequent investigation, and the tomato possessing a high Cd uptake rate was chosen as the remediation plant (Zhao et al. 2015). This work investigates the impacts of energy production in *B. amyloliquefaciens* Bam1 under Cd stress on Cd phytoremediation by tomatoes. It provides a more comprehensive understanding of the mechanisms of bioremediation technology in heavy metal pollution control and promotes product development with the Bam1 strain in Cd bioremediation.

## Materials and methods

### Strains and mutant strains construction

*B. amyloliquefaciens* wild-type strain Bam1 (CGMCC 21633) used in this study were obtained from the suburban area of Beihai, Guangxi, China.

The strains and plasmids used in this study are listed in Table S1. The primers used in this study are listed in Table S2, and PCR conditions are detailed in Table S3.

The recombinant plasmids were constructed by seamless cloning kit (HB-infusionTM, Hanheng Biotechnology Co., Ltd). The genome of *B. amyloliquefaciens* Bam1 was used as the templates for PCR. The *sdhA*, *fumC* and *qoxD* from *B. amyloliquefaciens* Bam1 were amplified by PCR with specific primers in Table S2. The recombinant plasmids were demethylated by the *Escherichia coli* GM2163 strain and then transferred into the Bam1 strain by electroporation to construct the mutant strains.

### Media and culture conditions

#### Media

The culture medium for strain construction and normal strain culture was Luria–Bertani (LB) broth (of per liter: tryptone 10 g, yeast extract 5 g, sodium chloride 10 g). Double-layer LB medium or Minimal medium (MM medium (Que and Helmann 2000), containing 20 g/L malic acid as a carbon source) was used to evaluate cadmium resistance. Hard (bottom) and soft (top) agars for double-layer medium were prepared by adding 1.5% agar or 0.75% (w/v) agar to LB medium or MM medium, respectively. Appropriate antibiotics were included at the following concentrations: ampicillin 100 mg/L and tetracycline 20 mg/L.

#### Culture conditions

A single fresh colony on the solid LB-medium was inoculated in 4 mL of LB liquid medium and at 37 °C with 220 rpm for overnight. The preculture was inoculated (2%, v/v) into 250-mL shaking flask containing 50 mL Minimal medium for aerobic growth at 220 rpm and 37 °C for 10 h. The secondary culture was inoculated into 50 mL (2% v/v) of Minimal medium at 37 °C, 220 rpm, with or without 500 nM CdCl_2_, according to the treatment.

### Cd resistance evaluation

#### Disk diffusion assay

When evaluating the ability of a strain to resist Cd on solid media, a disk diffusion assay was employed. The tested single colony was inoculated into 4 mL of LB liquid and cultured in a shaking flask to an OD_600_ of 0.8. 100 μL of bacterial culture broth was mixed evenly with 4 mL of soft agar at approximately 55 °C, then poured onto the prepared hard agar. Following the solidification of the double-layer agar, a piece of sterilized filter paper was placed on the agar, and 10 μL of a 10 mM CdCl_2_ solution was added to the filter paper. After incubating at 37 °C for 18 h, the diameter of the inhibition zone for each treatment was recorded.

#### Growth curve assay

When evaluating the ability of a strain to resist Cd in liquid media, a growth curve assay was used. After activated on solid LB agar and cultured in LB broth, the secondary culture was inoculated into 50 mL (2% v/v) of Minimal medium at 37 °C, 220 rpm, with 500 nM CdCl_2_. The growth curve was record and the maximum OD_600_ determined the Cd-resistance of the strain.

### Transcriptomic analysis

A transcriptomic analysis was conducted under Cd stress between the wild-type Bam1 and a Cd efflux pump deletion strain, Bam1Δ*cadA*. Bam1 and Bam1Δ*cadA* were cultured in 50 mL culture media containing 500 nM CdCl_2_ for 20 h (exponential growth phase) at 37 °C with 220 rpm. The cell pellets were collected by centrifuge (4 °C, 5500 rpm, 5 min) and stored at − 80 °C. After RNA extraction (Vazyme, Nanjing, China), transcriptome sequencing was conducted by Shenzhen Weike Meng Technology Group Co., Ltd (Shenzhen, China). Raw read lengths were obtained by sequencing on the Illumina platform NovaSeq 6000. Differential analysis was performed using EDGE-pro software.

A RT- qPCR analysis was used to validate the transcriptome analysis results. The RT- qPCR reaction mix and reaction procedures were listed in Tables S4, S5. Data were analyzed using method 2^−ΔΔCT^ and triplicates were run in each RT-qPCR reaction.

### Colonization in soil

The colonization experiments in Cd-contaminated soil (containing 5 mg/kg Cd, collected from tomato planting field in Zhuzhou suburbs, Hunan, China) was conducted with the wild-type Bam1 and the energy production recombinant strains Bam1*sdhA*, Bam1*fumC*, and Bam1*qoxD*. Bam1Δ*cadA* inoculated in Cd-contaminated soil was employed as control treatment 1, and Bam1 inoculated in conventional soil was employed as control treatment 2. After activation from − 80 °C, all the strains were culture in LB (recombinant strains culture in LB with tetracycline) until the OD_600_ reach ~ 1.0. Then 20 mL broth of each tested strain was mixed with 100 g sterilized soil (details of soil sterilization were listed in “Supplementary Material”). The bacteria population in the soil of each treatment was around 10^7^ cfu/g at the beginning. All the treatments were cultured at 30 °C, and the population of the living bacteria of each treatment was determined on the 0th, 1st, 3rd, 5th, 7th, 11th and 17th day. Each treatment contained three replicates.

### Pot test design

Tomato (*Solanum lycopersicum* Hezuo 903) with relatively strong Cd uptake ability was used as remediation plant. Tomato seedlings grown to the "two-leaf stage" were transplanted to conventional nutrient soil (control, no Cd contamination, containing organic matter, meeting the Chinese National Grade Standard (GB/T 33891-2017) for organic nutrient soil.) and Cd-contaminated soil containing 5 mg/kg Cd (a 2.5 g/L CdCl_2_ stock solution was prepared, diluted and mixed with conventional nutrient soil to achieve a terminal concentration of 5 mg Cd/kg soil). On the third day after transplanting, the roots of the treated plants were drenched with 15 mL of the culture of Bam1 and its mutant strains (containing approximately 5 × 10^7^ CFU/mL) and the same amount of water was added as a control. The conventional nutrient soil treatment groups were Water, Bam1, Bam1*sdhA*, Bam1*fumC*, and Bam1*qoxD*, and the Cd-contaminated soil treatment groups were Cd + Water, Cd + Bam1, Cd + Bam1*sdhA*, Cd + Bam1*fumC*, and Cd + Bam1*qoxD*. Chlorophyll content and plant height were examined regularly, and all samples were collected after 14 days for determination of root length and Cd content. There were three replicates of each treatment and 10 plants in each replicate.

### Analytical methods

#### Cell density measurement

Cell density was measured by the optical absorbance at 600 nm (OD_600_) with appropriately diluted culture samples.

#### ATP and ROS measurement

The pipette strain cells were washed twice with PBS solution and resuspended to measure the cells’ amount of ATP and ROS. The amount of ATP in cells was determined using the ATP assay kit (Beyotime, Jiangsu, China), according to the manufacturer’s protocols. ROS levels were tested using a fluorometric intracellular ROS kit (Beyotime, Jiangsu, China), according to the manufacturer’s protocols. The fluorescence was measured by plate reader (Synergy H1, BioTek, USA).

#### Chlorophyll content determination in plant

Chlorophyll was determined using a Chlorophyll meter (Spad-502Plus, Konica Minolta, Japan). All leaves of each plant for each treatment were tested three times and the means were compared with other treatments for significance.

#### Cd content determination in plant

When the pot test was finished, each plant was divided into above-ground parts (stems and leaves) and below-ground parts (roots), washed with deionized water and dried at 105 °C for 30 min and then at 70 °C for 3 days to determine dry biomass. The Cd concentration in plant tissues were detected according to Xiong et al. with modification (Xiong et al. 2022). Ground plant tissues (1.0 g) were digested with HNO_3_ (3%) in a Microwave Digestion System (Multiwave 7000, Anton Paar, Austria), then brought up to 25 mL with HNO_3_ (3%). The Cd concentration in the digests were determined by ICP-MS (NexION 2000, PE, USA).

### Statistical analysis

A minimum of three independent biological replicates were performed in all experiments. For significant differences among different treatments, one-way analysis of variance (ANOVA) was carried out with GraphPad Prism (version 8, GraphPad Software, USA). A t-test was performed to compare amounts between groups.

## Results and discussion

### Transcriptome analysis of Bam1 under Cd stress

**Fig. 1:**
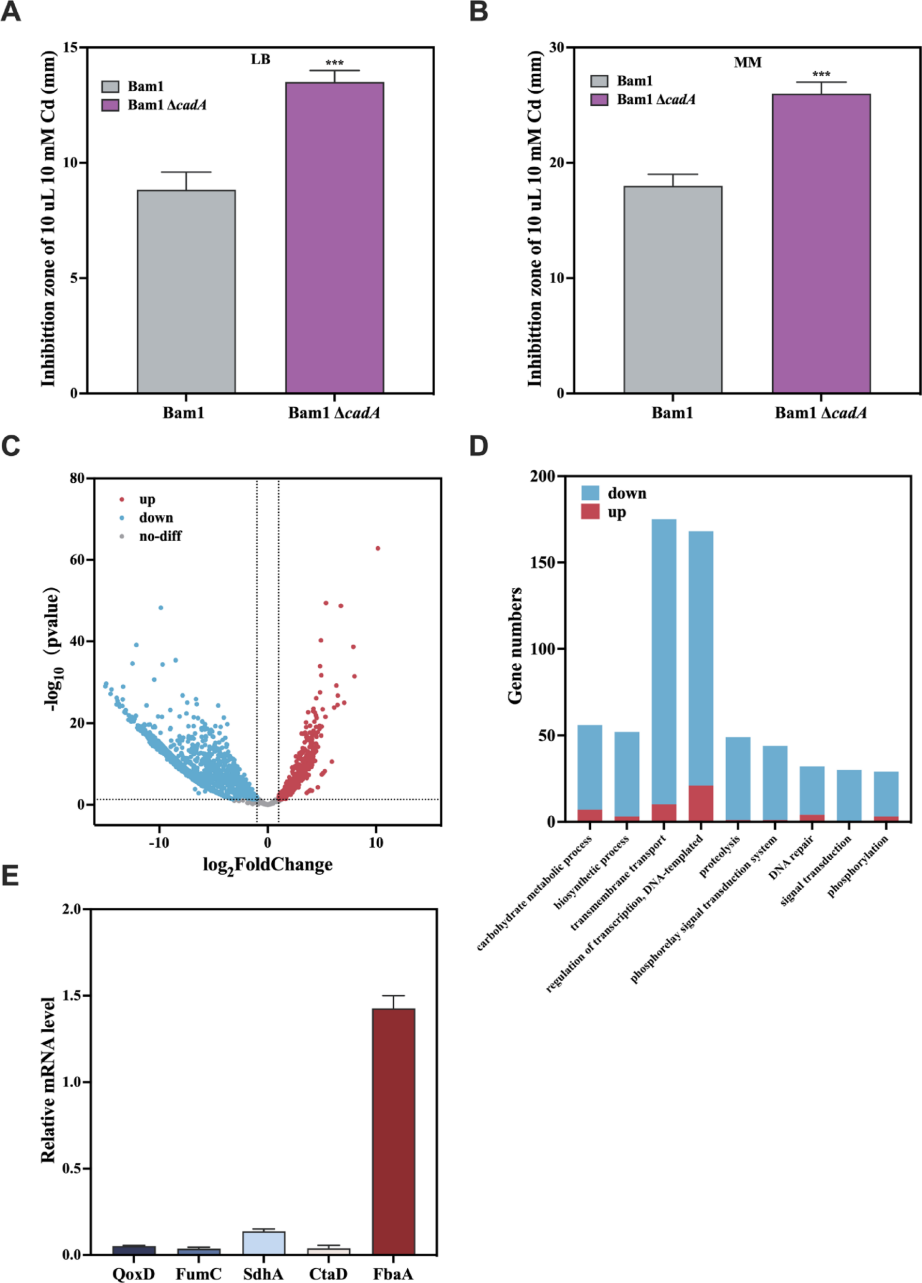
Bam1 and Bam1Δ*cadA*’s Cd resistance evaluation and transcriptome analysis. Inhibition zone diameters of Bam1 and Bam1Δ*cadA* in LB medium (**A**) and MM medium (**B**), summary of upregulated and downregulated genes of Bam1Δ*cadA* compared to Bam1 (**C**), differential gene expression of Bam1Δ*cadA* compared to Bam1 (**D**) and gene expression levels of Bam1Δ*cadA* compared to Bam1 (**E**). Significance analysis of differences between Bam1Δ*cadA* and Bam1, ***significant difference at *p* < 0.001

*B. amyloliquefaciens* Bam1 strain possessed excellent Cd resistance, and the Cd efflux pump, CadA, significantly affected its Cd resistance (Luo et al. 2022). When the *cadA* gene was deleted from Bam1 strain, the Cd resistance of Bam1Δ*cadA* decreased sharply on both rich medium (LB) and minimal medium (Fig. 1A and B). While on the nutrition-limited Minimal medium, Bam1Δ*cadA* was more sensitive to Cd than on the nutrition-rich LB medium (Fig. 1A and B). These results align with those of Chmielowska’s study (Chmielowska et al. 2021), suggesting energy production may be a critical influence on the Cd resistance of Bam1.

In order to ascertain the pivotal genes that influence the Cd resistance of Bam1, a transcriptomic comparison was conducted between Bam1 and Bam1Δ*cadA* under Cd stress (500 nM Cd). Compared to the Bam1 strain, the Bam1Δ*cadA* strain exhibited a greater number of down-regulated genes (3513) than those up-regulated (396) in response to Cd stress (Fig. 1C). Among the significant up and down-regulated genes, the three biological processes with the greatest differences in gene expression were transmembrane transport, gene transcription regulation, and carbohydrate metabolism (Fig. 1D). For validation of the transcriptome sequencing results, five genes were randomly selected for RT-qPCR analysis in Bam1 and Bam1Δ*cadA*. The expression levels of *fumC*, *sdhA*, *ctaD* and *qoxD* genes in Bam1Δ*cadA* were lower than those in Bam1, while the expression level of *fbaA* gene was higher in Bam1Δ*cadA* compared to Bam1, which was consistent with the transcriptome sequencing results (Fig. 1E). This indicates that the transcriptome sequencing results were accurate and can serve as a basis for analyzing the Cd resistance mechanism.

**Fig. 2:**
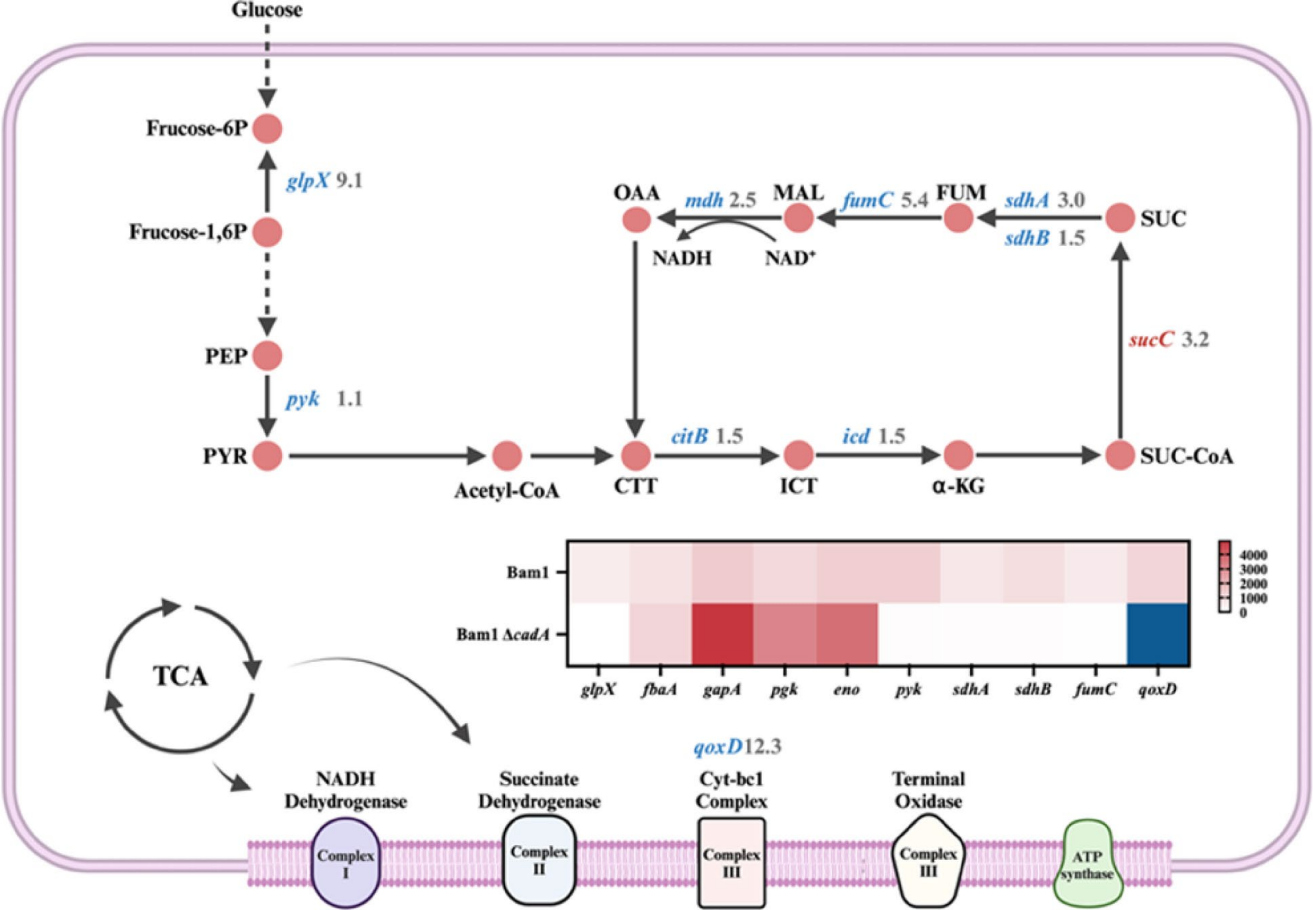
Differential gene expression in the main energy production metabolic pathways of Bam1Δ*cadA* compared to Bam1. Fructose-6-P: fructose-6-phosphate; Frutose-1,6-P: fructose-1,6-diphosphate; PEP: phosphoenolpyruvate; PRY: pyruvate; CTT: citrate; ICT: isocitrate; α-KG: α-Ketoglutarate; SUC-CoA: succinate-CoA; SUC: succinate; FUM: fumarate; MAL: malate; OAA: oxaloacetate; *glpX*: fructose 1,6-bisphosphatase; *pyk*: pyruvate kinase; *citB*: aconitate hydratase; *icd*: isocitrate dehydrogenase; *sucC*: succinyl-CoA synthetase; *sdhAB*: succinate dehydrogenase; *fumC*: fumarase; *mdh*: malate dehydrogenase; *qoxD*: cytochrome aa3 quinol oxidase. Red indicates up-regulated genes, blue indicates down-regulated genes

With regard to carbohydrate metabolism, a decrease in expression was observed for 49 genes, while 7 genes demonstrated an increase. The findings provided scientific evidence to support the assertion that the Bam1Δ*cadA* strain exhibits a limited capacity to resist the impact of Cd, which was consistent with the result in Fig. 1A, B. This suggests that the carbon metabolism pathway involved in energy production considerably influences Bam1’s Cd resistance. Therefore, further comparative transcriptomic analysis was performed on the main pathways involved in energy production in these two strains, the central carbon metabolism and the respiratory chain pathways (Fig. 2).

In the glycolysis pathway, it was observed that the genes *gapA*, *pgk*, and *eno* were all up-regulated about 4-fold in Bam1Δ*cadA* under Cd stress compared to the Bam1 strain. The *gapA* gene encodes glyceraldehyde-3-phosphate dehydrogenase (GAPDH), which is an intracellular sensor of oxidative stress during early apoptosis and the overexpression of GAPDH can lead to apoptosis. The entire GapA operon comprises the genes *gapA*, *pgk*, and *eno* (Jannière et al. 2007), so it is speculated that the up-regulation of these three genes under Cd stress leads to cell death. The *fbaA* gene (encoding fructose-1,6-bisphosphate aldolase) in Bam1Δ*cadA* was also up-regulated 4 times. The *fbaA* gene has also been reported to be involved in spore formation (Endo and Kurusu 2007), which is important for the survival and stress resistance of *Bacillus*. The up-regulation of *fbaA* suggests that under Cd stress, Bam1Δ*cadA* produces more spores to resist the stress. However, spore production is not sufficient to compensate for the energy loss, resulting in insufficient growth overall and thus a reduction in Cd resistance of Bam1Δ*cadA*. The expression level of the *pyk* gene decreased 1.1-fold in Bam1Δ*cadA*, which encodes pyruvate kinase to catalyze the production of the end product of glycolysis, pyruvate. This indicated a reduction in the carbon flux to the TCA cycle, resulting in an insufficient supply of energy for cell growth and division, thus reducing Cd resistance. In the TCA cycle, the expression levels of *sdhA* (encoding succinate dehydrogenase flavoprotein subunit A), *sdhB* (encoding succinate dehydrogenase flavoprotein subunit B), and *mdh* (encoding malate dehydrogenase) genes were down-regulated by 3.0-fold, 1.5-fold, and 2.5-fold, respectively, under Cd stress, indicating a corresponding decrease in carbon flux to the respiratory chain. A deficiency in cellular ATP production resulting from a diminished carbon flux to the respiratory chain has been observed to impair the growth and resistance of the Bam1Δ*cadA* strain in the presence of Cd stress. The down-regulation of the majority of genes within the respiratory chain (e.g. *qoxD* encoding cytochrome aa3 quinol oxidase subunit IV, down-regulated by 12.3-fold) also suggested that Cd stress impeded the supply of energy required for cellular growth, thereby influencing the resistance of Bam1Δ*cadA* to Cd.

Most of the previous research on the mechanism by which PGPR promote Cd contamination remediation has focused on enhancing the strain’s own Cd efflux system (Chi et al. 2020); immobilizing Cd with siderophores, carbonic acid, organic acids, amino acids and chelating peptides produced by PGPR (Gupta et al. 2024); Cd adsorption by PGPR biofilms (Kaushal and Pati 2025), and promoting plant growth under Cd stress by producing plant hormones such as IAA ect. (Sharma et al. 2024a). Based on these principles, a few studies have found that genetically engineered strains can promote the remediation of Cd pollution. For example, in a *P. aeruginosa* (Pse-w) isolated from a Cd-contaminated oil field, the expression of metallothionein was enhanced on the surface of Pse-w to develop the Psew-MT engineered strain (Gupta et al. 2024). This promoted Cd immobilization by absorbing more Cd into the metallothionein (Gupta et al. 2024). Currently, PGPR primarily includes strains of *Bacillus* that are used for the remediation of Cd contamination, either individually or in combination with accumulator plants, examples include *B. subtilis*, *B. cereus*, and *B. megaterium* (Chi et al. 2020; Kaushal and Pai 2025), among others. However, there is relatively little research on *B. amyloliquefaciens*, and the anti-Cd mechanisms of these strains have not yet been fully investigated. A few omics-stage studies have found that energy changes could be a potential mechanism by which PGPR promotes its own Cd resistance (Khan et al. 2017; Zhao et al. 2025a, b). However, there is a lack of corresponding validation experiments.

In summary, there are currently very few reports on enhancing the Cd resistance of PGPR through energy related pathways. Furthermore, no research has been conducted on improving the Cd resistance of PGPR strains through genetically modified energy-related pathways in PGPR. Based on the above transcriptome analysis, several key genes in the energy production pathway found may significantly impact the energy level of Bam1. Therefore, the recombinant strains of Bam1 involved in energy production pathways would be constructed in order to verify the effect of these genes on the Cd resistance of Bam1.

### Effect of key energy production genes on promoting Cd resistance in Bam1

**Fig. 3:**
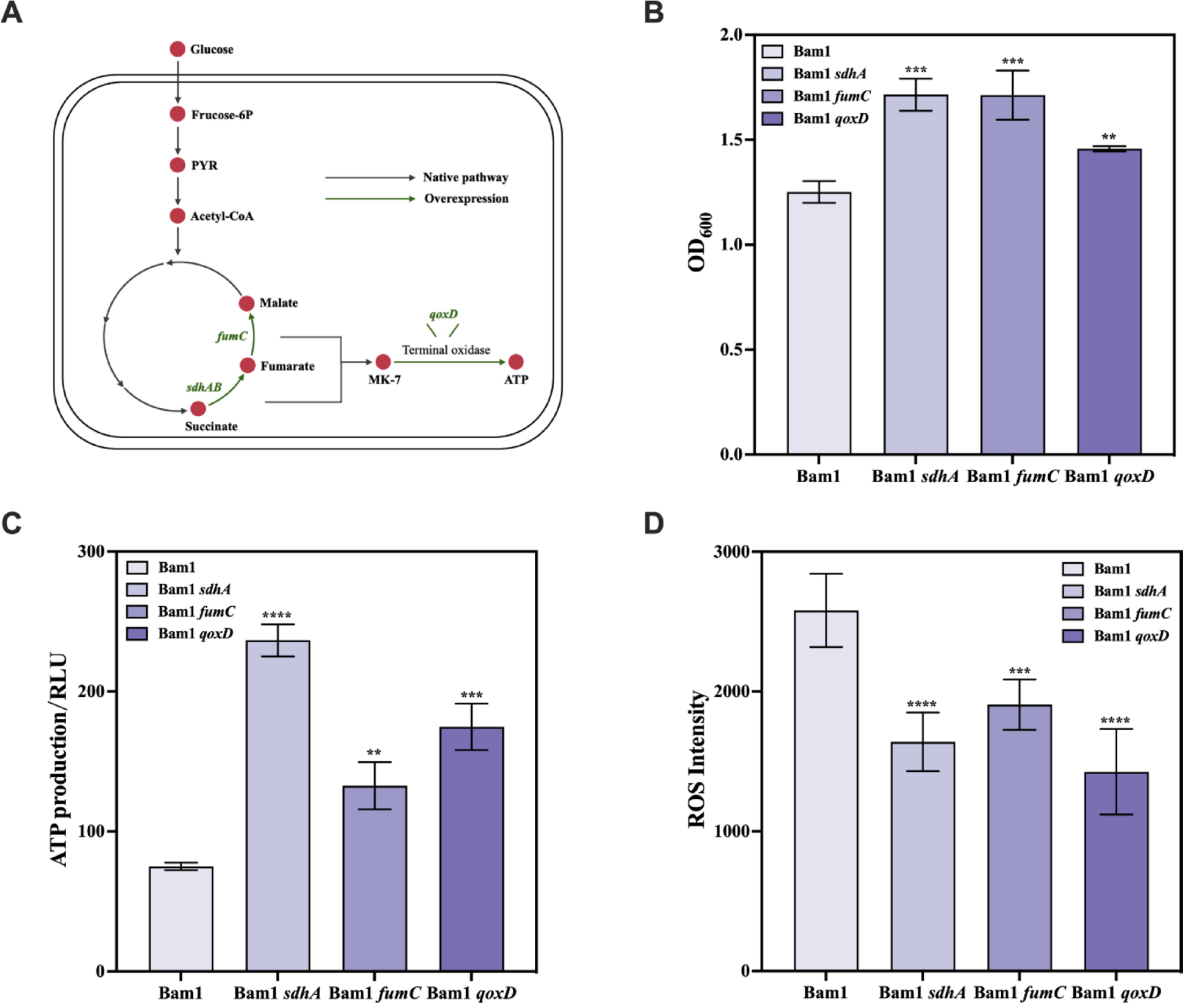
Recombinant strains’ growth under Cd stress and production of energy and ROS. Genetic manipulation performed in this step (**A**), maximum OD_600_ values of recombinant strains compared to Bam1 (**B**), ATP production (**C**) and ROS production (**D**) of recombinant strains compared to Bam1 at maximum OD_600_ values. Significance analysis of differences between each recombinant strain treatment compared to Bam1; **, ***, and ****: significant difference at *p* < 0.01, *p* < 0.001, and *p* < 0.0001 level, respectively

According to the transcriptome analysis, the key genes of energy production (*sdhA*, *fumC*, *qoxD*) in Bam1Δ*cadA*, were significantly down-regulated under Cd stress compared to Bam1 strain. To verify the effect of the aforementioned genes on the resistance of Bam1 to Cd, *sdhA*, *fumC*, *qoxD* were overexpress individually in Bam1 and named as Bam1*sdhA*, Bam1*fumC*, and Bam1*qoxD*, respectively (Fig. 3A). These recombinant strains (Bam1*sdhA*, Bam1*fumC*, and Bam1*qoxD*) and wild-type strain (Bam1) were then cultured in MM medium with 500 nM Cd. The maximum OD_600_ value demonstrated that all three recombinant strains significantly promoted their Cd resistance in comparison with the wild-type Bam1 strain. The maximum OD_600_ of the recombinant strains were 1.72, 1.71, and 1.46, however, the maximum OD_600_ of Bam1 just reached 1.25 (Fig. 3B). Further investigation of energy production and ROS levels in cells at maximum OD_600_ showed that all three recombinant strains produced more energy but less ROS compared to the wild-type Bam1 strain. ATP production of Bam1*sdhA*, Bam1*fumC* and Bam1*qoxD* was approximately 3.15, 1.77 and 2.33 times that of the Bam1 (Fig. 3C). While the ROS level in Bam1*sdhA*, Bam1*fumC*, and Bam1*qoxD* was only about 63.55%, 73.85% and 55.28% that of the Bam1 (Fig. 3C, D). These results suggested that the overexpression of genes *sdhA and fumC* improved the carbon flux to the respiratory chain, and the overexpression of gene *qoxD* improved the efficiency of the electron transport chain. Then resulted in increased energy production and reduced the generation of harmful ROS (Agrawal et al. 2017; Liu et al. 2024; Weidner et al. 1993), ultimately enhancing Bam1’s resistance to Cd. Khan et al. utilized proteomic analysis to demonstrate that carbohydrate metabolism was the potential mechanism of the *E. coli* P4 strain in coping with Cd stress (Khan et al. 2017). Here, we confirmed a comparable mechanism in *B. amyloliquefaciens* Bam1 strain by overexpressing the pivotal enzymes involved in energy production.

### Effect of key energy production genes on enhancing Bam1’s colonization in Cd-contaminated soil

**Fig. 4:**
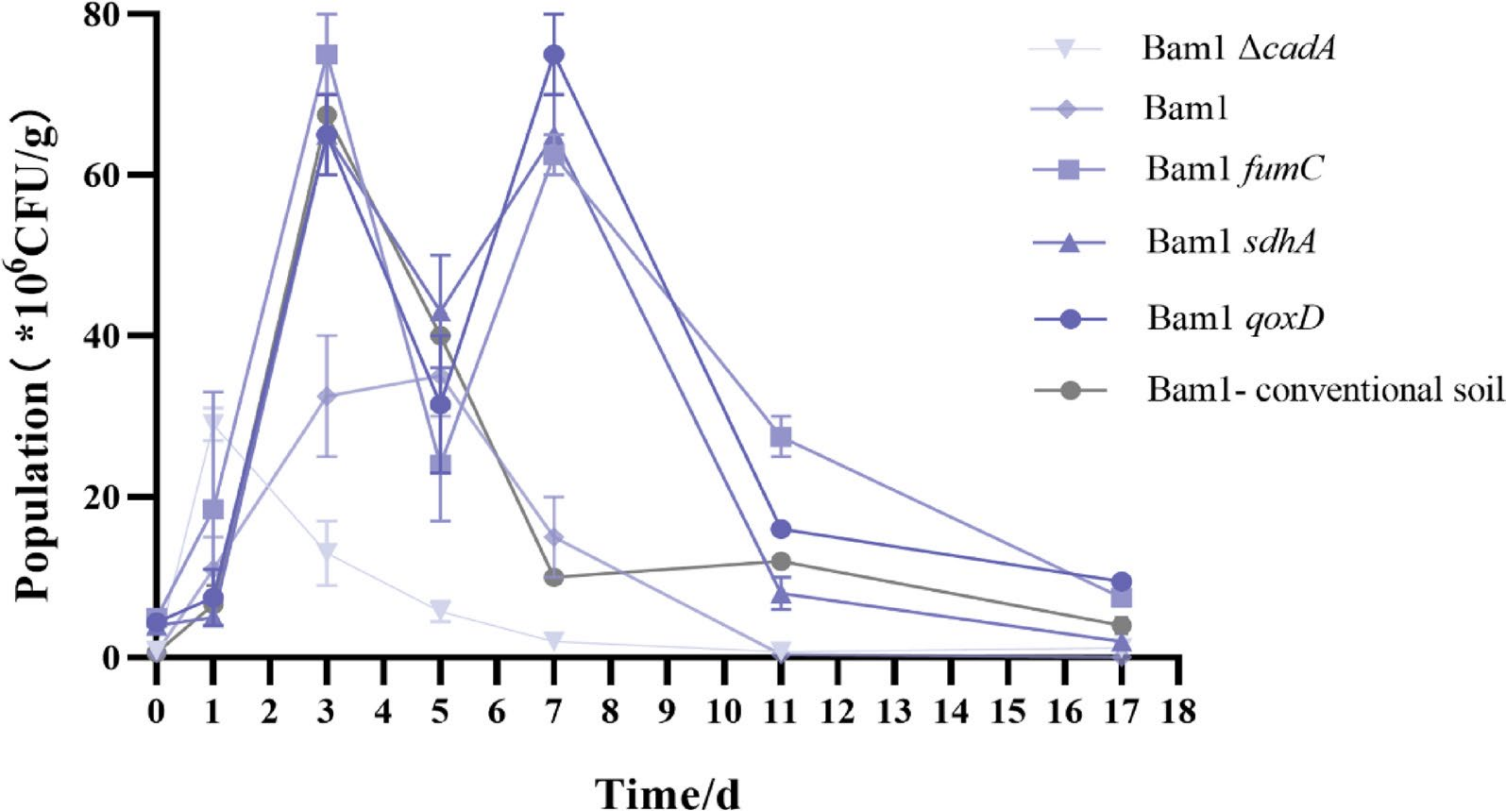
Effect of Bam1 and its recombinant strains on the colonization in Cd-contaminated soil (5 mg/kg)

Though Bam1*sdhA*, Bam1*fumC*, and Bam1*qoxD* showed stronger Cd resistance than Bam1 in MM liquid medium, colonization in the application niches is the primary determinant of the PGPR’s ability to act as a plant growth promoter (Wang et al. 2021b). The colonization of the three recombinant strains (Bam1*sdhA*, Bam1*fumC*, and Bam1*qoxD*), the wild-type Bam1 strain and the *cadA* deleted strain (Bam1Δ*cadA*) were further investigated in soil with 5 mg/kg Cd. Bam1 colonization in conventional nutrient soil as control. The results (Fig. 4) showed that all strains exhibited an increase in population following adaptation. Bam1Δ*cadA* exhibited the least efficient colonization in Cd-contaminated soil (the maximum population was 2.9 × 10^7^ CFU/g). Bam1’s colonization was superior to that of Bam1Δ*cadA* in the same condition (the maximum population was 3.5 × 10^7^ CFU/g). Whereas the three recombinant strains exhibited the most effective colonization in Cd-contaminated soil (the maximum population were 6.5 × 10^7^ CFU/g, 7.5 × 10^7^ CFU/g and 7.5 × 10^7^ CFU/g respectively), even surpassing the colonization of Bam1 in conventional soil after 7 days of inoculation (Fig. 4). Among the three recombinant strains, the colonization of Bam1*fumC* and Bam1*qoxD* was better than that of Bam1*sdhA* at the later phase (after 11 days of inoculation). The trends observed in the colonization of all these strains were consistent with their resistance to Cd in MM medium, indicating that enhancing the Cd resistance of strains can improve their colonization and reproduction in Cd-contaminated soil.

### Effect of energy boosting strains on promoting plant resistance to Cd stress

The energy production recombinant strains (Bam1*sdhA*, Bam1*fumC*, and Bam1*qoxD*) could effectively colonize in Cd-contaminated soil. To ascertain whether they could also enhance the plants’ Cd resistance, an investigation was conducted into the changes in chlorophyll content, plant height, and root length of tomatoes drenched with Bam1 and its recombinant strains in soil with (5 mg/kg Cd) or without Cd. Water treatment as a control. This investigation was undertaken to explore their capacity to enhance tomatoes’ photosynthetic capabilities and growth in soil with or without Cd.

**Fig. 5:**
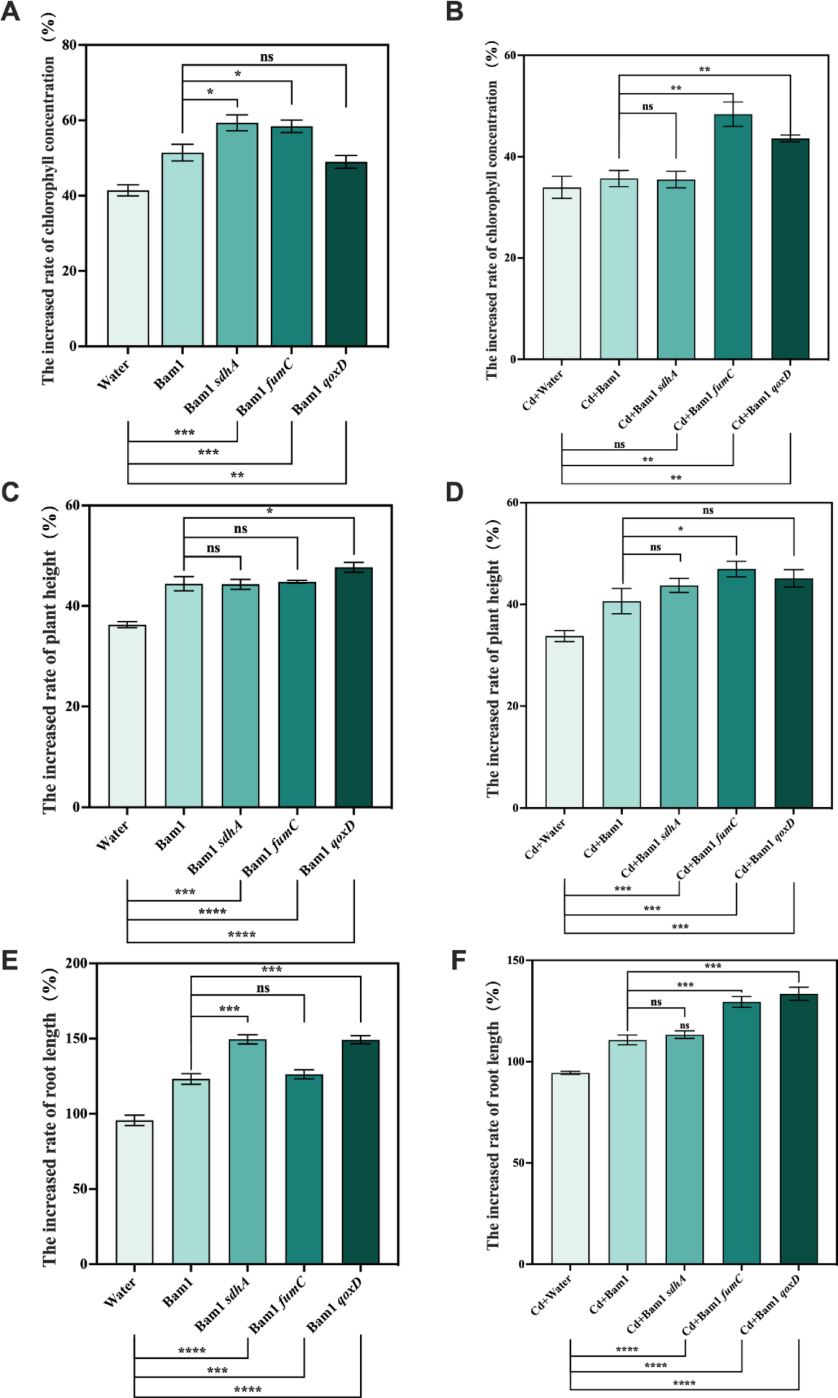
Effect of Bam1 and its recombinant strains on the chlorophyll concentration, plant height, and root length of tomato seedlings. The increased rate of chlorophyll concentration of tomatoes grown in the conventional nutrient soil (**A**) and Cd-contaminated soil (**B**); the increased rate of plant height of tomatoes grown in conventional nutrient soil (**C**) and Cd-contaminated soil (**D**); the increased rate of root length of tomatoes grown in conventional nutrient soil (**E**) and Cd-contaminated soil (**F**). Significance analysis of differences between each recombinant strain treatment and Bam1 WT, or water control; ns: the difference is not significant; *, **, ***, ****: significant difference at *p* < 0.05, *p* < 0.01, *p* < 0.001, *p* < 0.0001 level, respectively

In comparison with the control group (Water), the application of Bam1 and its energy production recombinant strains resulted in a great enhancement of tomato photosynthesis and growth, both in soil with and without Cd (Fig. 5A–F). The improvements in conventional soil treatments (Bam1, Bam1*sdhA*, Bam1*fumC*, and Bam1*qoxD*) were found to be more pronounced than those in Cd-contaminated soil treatments (Cd + Bam1, Cd + Bam1*sdhA*, Cd + Bam1*fumC*, and Cd + Bam1*qoxD*). It was suggested that the presence of Cd resulted in a suppression of tomato photosynthesis and growth, while the application of Bam1 and its energy production recombinant strains could mitigate this stress.

When compared with Bam1’s treatment in Cd-contaminated soil (Cd + Bam1), the Cd + Bam1*fumC* and Cd + Bam1*qoxD* treatments significantly increased chlorophyll concentration in tomato seedlings’ leaves (the increased rate of chlorophyll concentration was 1.35 and 1.22 times that of the Cd + Bam1 treatment), while the Cd + Bam1*sdhA* treatment exhibited no significant increase of chlorophyll concentration (Fig. 5B). The plant height of tomatoes in the Cd + Bam1*fumC* treatment was found to be significantly increased in comparison with the Cd + Bam1 treatment (the increased rate of plant height was 1.16 times that of the Cd + Bam1 treatment). In contrast, the plant height of tomatoes in the Cd + Bam1*qoxD* and Cd + Bam1*sdhA* treatments was only increased slightly and not significantly when compared with the Cd + Bam1 treatment (Fig. 5D). In comparison with the Cd + Bam1 treatment, the root length of tomatoes in the Cd + Bam1*fumC* and Cd + Bam1*qoxD* treatments exhibited a significant increase (the increased rate of root length was 1.13 and 1.21 times that of the Cd + Bam1 treatment). However, this increase was not observed in the Cd + Bam1*sdhA* treatment. (Fig. 5F). In conclusion, Bam1*fumC* strain significantly enhanced the photosynthesis and growth of tomatoes under severe Cd stress (5 mg/kg). Despite Bam1*sdhA* strain demonstrating the highest energy production among the three recombinant strains, its colonization efficacy was the least effective at the later stage. Conversely, Bam1*fumC* strain exhibited the most effective colonization at the later stage. It can be deduced that the primary factor influencing the promotion effect of alleviating Cd stress in remediation plants is the colonization of PGPR. Zhang et al. (2013) also found that the effective colonization of endophytic bacteria (*Sphingomonas* sp.) can significantly enhance the growth of the remediation plants, *Sedum alfredii*, in Cd-contaminated soil.

### Effect of energy boosting strains on promoting Cd phytoremediation

Bam1 and its recombinant strains, especially the Bam1*fumC* strain, could alleviate the Cd stress and improve the photosynthesis and growth of tomatoes in Cd-contaminated soil. However, we wondered if they could also improve the phytoremediation of tomatoes for Cd contamination. Therefore, in the final stage of the pot test, the Cd uptake of the above- and below-ground tissues of tomatoes was measured.

**Fig. 6:**
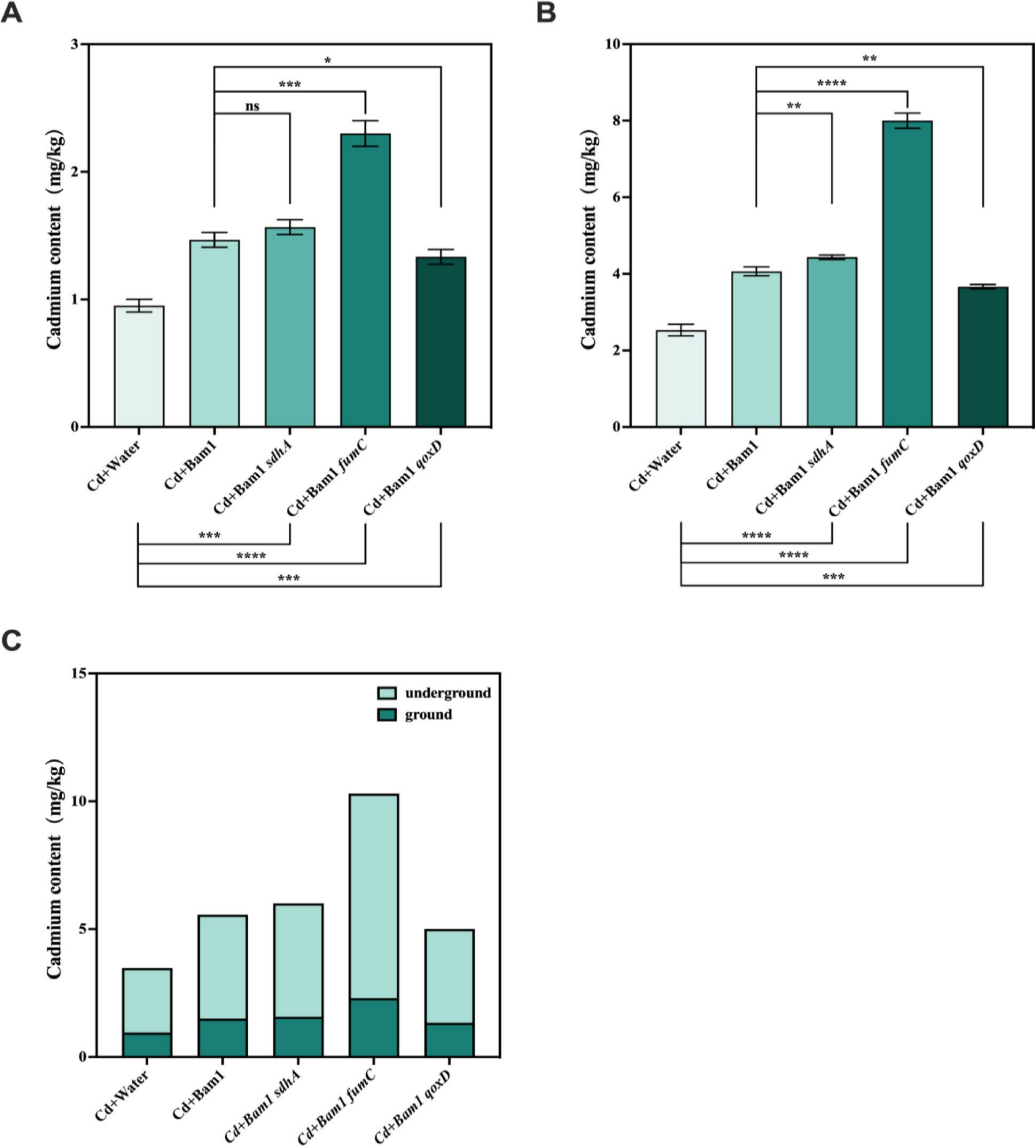
Effect of Bam1 and its recombinant strains on Cd absorption of tomato seedlings in Cd-contaminated soil. Above-ground part of tomato seedlings (**A**), underground part of tomato seedlings (**B**) and addition of cadmium uptake by above-ground and below-ground parts of tomato seedlings of different bacterial strains (**C**). Significance analysis of differences between each recombinant strain treatment and Bam1 WT, or water control; ns: the difference is not significant; *, **, ***, ****: significant difference at *p* < 0.05, *p* < 0.01, *p* < 0.001, *p* < 0.0001 level, respectively

In comparison with the control treatment (Cd + Water), Bam1 and its reconstructed strains exhibited a marked enhancement in the accumulation of Cd by tomatoes (Fig. 6). This finding suggested that these strains possessed the capacity to substantially augment the efficiency of tomatoes in phytoremediation in Cd-contaminated soil. The Cd content in the above-ground parts of tomato plants in Bam1 and its recombinant strains treatment increased by 26.32–152.63% in comparison with the control treatment (Fig. 6A). Meanwhile, the content of Cd in the below-ground parts of tomato plants in the Bam1 and its recombinant strains treatment increased by 44.74–215.79% in comparison with the control treatment (Fig. 6B). When the Cd content of the above-ground and below-ground parts was compared, all treatments showed higher Cd accumulation in the under-ground parts (Fig. 6C), indicating that most of the Cd was immobilized in the root of tomatoes. Li et al. also obtained analogous results in their study on the utilization of *B. subtilis* to enhance ryegrass’s capacity for phytostabilization of Cd-contaminated soil (2022). The relatively low Cd content in the above-ground part is beneficial for plants, as it helps them maintain normal photosynthesis and produce the necessary nutrients for growth.

A comparison of the three recombinant strain treatments revealed that Cd + Bam1*fumC* exhibited the highest Cd accumulation in both the below-ground and above-ground parts, significantly exceeding the levels observed in the other treatments (Fig. 6). This finding suggested that Bam1*fumC* had the optimal capacity to promote Cd accumulation in tomatoes (the Cd accumulated in tomatoes in the Cd + Bam1*fumC* treatment was 1.88 times that in the Cd + Bam1 treatment, and 2.96 times that in the control treatment). Although the Cd + Bam1*qoxD* treatment accumulated more Cd than the control treatment (Cd + Water), this was less than the Cd + Bam1 treatment. However, its effects on chlorophyll content, plant height, and root length were superior to those of the Cd + Bam1 treatment. This suggested that while Bam1*qoxD* enhanced tolerance to Cd and improved plant growth, it may also affect the ability to accumulate Cd in tomatoes. Some studies suggest that *B. amyloliquefaciens* can promote the hemicellulose content in *Arabidopsis* cell walls, leading to more Cd fixation in the cell wall (Zhou et al. 2017). Whether Bam1*qoxD* affects these properties of *B. amyloliquefaciens* and then affects the Cd absorption and fixation capabilities in plants remains to be elucidated through further investigation. The comprehensive results of the colonization and pot tests indicate that Bam1*fumC* was the optimum energy production recombinant strain. This strain could efficiently colonize in Cd-contaminated soil, thereby promoting tomato growth, and ultimately significantly enhancing the phytoremediation of tomatoes for Cd pollution.

## Conclusion

The pivotal genes implicated in energy production in Bam1 (*sdhA*, *fumC*, and *qoxD*) were selected to construct the energy production recombinant strains, after comparative transcriptome analysis under Cd stress. The recombinant strains increased their Cd resistance by producing more ATP and less ROS, and thus better colonized in Cd-contaminated soil. With the excellent colonization in Cd-contaminated soil, the optimum energy recombinant Bam1*fumC* significantly improved the photosynthesis and growth of tomato, and ultimately resulted in an efficient enhancement in the phytoremediation of tomatoes on Cd. The Cd accumulated in tomatoes in the Cd + Bam1*fumC* treatment was 1.88 times that in the Cd + Bam1 treatment, and 2.96 times that in water control treatment. Bam1*fumC* exhibited the considerable potential for development as a bioaugmentation assistant in Cd-contaminated phytoremediation. Further field trials should be conducted to accelerate its product development. Multi-metal stress testing should also be studied to broaden its applications. This study provided insights into the mechanisms of energy metabolic pathway on the influence of microbial Cd resistance and Cd contamination phytoremediation and thus offers a novel strategy for addressing soil Cd pollution remediation.

## Supplementary Information

Below is the link to the electronic supplementary material.

Supplementary Material 1

## Data Availability

Not applicable.
