# Supplementary material for: Impact of energy metabolism pathways in promoting phytoremediation of cadmium contamination by Bacillus amyloliquefaciens Bam1
Source: Bioresour Bioprocess. 2025 Nov 10;12(1):134. doi: 10.1186/s40643-025-00972-8 (PMC12597858; doi:10.1186/s40643-025-00972-8)
Supplement: Supplementary file 1 — Supplementary Material 1 [file 40643_2025_972_MOESM1_ESM.pdf]

SUPPLEMENTARY MATERIAL 1

Impact of energy metabolism pathways in promoting phytoremediation of cadmium contamination by *Bacillus amyloliquefaciens* Bam1

Xinting Jiang [1 #], Xiaomin Chen [1 #], Hongxia Gao [1], Jinyan Luo [2], Lin Zhang [3], Yuanchan Luo [1 *], Hui Wu [1, 4 *]

[1] State Key Laboratory of Bioreactor Engineering, Shanghai Collaborative Innovation Center for Biomanufacturing Technology, School of Biotechnology, East China University of Science and Technology, 130 Meilong Road, Shanghai 200237, China

[2] Department of Plant Quarantine, Shanghai Extension and Service Center of Agriculture Technology, Shanghai 201103, China

[3] School of Chemistry & Molecular Engineering and Research Centre of Analysis and Test, East China University of Science and Technology, Shanghai 200237, China

[4] MOE Key Laboratory of Bio-Intelligent Manufacturing, School of Bioengineering, Dalian University of Technology, Dalian, 116024, China

* Corresponding author: Yuanchan Luo

Telephone: +86-21-64252256

Fax: +86-21-64252250

E-mail: luoyuanc@ecust.edu.cn

* Corresponding author: Hui Wu

Telephone: +86-21-64252256

Fax: +86-21-64252250

E-mail: hwu@ecust.edu.cn

[#]Both authors contributed equally in this work.

Strains, plasmids, Primers, reaction mix and reaction procedures for recombinant strains reconstruction and RT-qPCR

Table S1 Strains and plasmids

Strains and plasmids	Relevant characteristics	Source
**Strains**		
Bacillus amyloliquefaciens Bam1	Wild type	Laboratory collection (CGMCC 21633)
Bam1Δ*cadA*	Laboratory collection	Laboratory collection
Escherichia coli GM2163	Wild type	Laboratory collection
**Plasmids**		
pT17	*E. coli – B. subtilis* shuttle vector with P43 promoter. Tet^r, Amp^r	Laboratory collection
pT17 *sdhA*	pT17 carries *sdhA* gene from Bam1	This study
pT17 *fumC*	pT17 carries *fumC* gene from Bam1	This study
pT17 *qoxD*	pT17 carries *qoxD* gene from Bam1	This study

Table S2 Primers

Name	Primer sequence (5′ to 3′)
qPCR *ctaD* -F	TTGCGGTTGCGACGATGGC
qPCR *ctaD* -R	GCGACGGCATACAGCATAGGC
qPCR *qoxD* -F	ATCCAAGTCGGAAACACGCTCTTC
qPCR *qoxD* -R	TCGTGATGTTCGGAATGCTGCTTC
qPCR *fumC* -F	TTGCCGCTGTATGTGACGATGTG
qPCR *fumC* -R	TGCTTTGTGTGCCGCTTCCC
qPCR *sdhA* -F	GGCGACAATTAAAGCAGCGGAATC
qPCR *sdhA* -R	CAGACCGAGTGAGAGCGTTTGAC
qPCR *fbaA* -F	GGCGGACAGGAAGATGACGTTATC
qPCR *fbaA* -R	AAGGACCGTGAACAGAACCCAATG
pT17 *sdhA* -F	AAGAGAGGAATGTACACATGAATATGAGTCAATCAAGCATTATTGTAGTCGGC
pT17 *sdhA* -R	CCTCTCTGCTCTTCCCGGGGATCTTATTTCGCCACCTTCTTCTTCGAGTAGT
pT17 *fumC* -F	AGAGAGGAATGTACACATGAATATGGACTACAGAATTGAAAAAGACACCATGG
pT17 *fumC* -R	CTCTCTGCTCTTCCCGGGGATCTTATGCTTTCGGATGCACCATATCTTCC
pT17 *qoxD* -F	AGAGAGGAATGTACACATGAATATGGCAAACAAATCTGCTGAACACAG
pT17 *qoxD* -R	CTCTCTGCTCTTCCCGGGGATCTTATTCGTGATGTTCGGAATGCTGCT
pT17 check -F	ATTTCGTGATGCTTGTCAGGGG
pT17 check -R	AATCCGTCCTCTCTGCTCTT

Table S3 PCR reaction procedures

Steps	Temperature	Times
1. Initial denaturation	98℃	5 min
2. Amplification	98℃	30 s
× 30 circle	55℃ (according to primers)	30 s
	72℃	2 kb/min
3. Final Extension	72℃	10 min

Table S4 RT-qPCR reaction mix

Components	Volume (μL)
5×One Step RT-qPCR buffer	4.0
Hot Start Taq DNA polymerase	0.2
Reverse transcriptase	0.4
Primers mix (Table S2)	2.0
TaqMan hydrolysis probe	0.4
RNA template	3.0
Nuclease-free H_2O	10.0
**Tataol**	**20.0**

Table S5 RT-qPCR reaction procedures

Steps	Temperature	Times
1. reverse transcription	55℃	15 min
2. Initial denaturation	94℃	15 min
3. Amplification	94℃	10 s
× 45 circle	58℃	35 s
	72℃	2 kb/min
4. Final Extension	72℃	5 min

(Touch CFX96TM, Bio-Rad, USA; Negative and positive controls were used in each run to evaluate the performance of the assay.)

Soil sterilization method

100g of soil was put in a 250 mL shake flask covered with paper to reduce water evaporation, then the soil was sterilized at 121℃ for 20 min. The sterilized soil was prepared for the colonization evaluation of Bam1 and its recombinant strains.
